# Methicillin-Resistant Staphylococcus aureus Bacteremia of Indeterminate Primary Source: A Case Report and Review of Pain Management During Hospital Course

**DOI:** 10.7759/cureus.25729

**Published:** 2022-06-07

**Authors:** Mariam Fatima, Wesley E Roach, Anvit D Reddy, Jeremy Olloqui

**Affiliations:** 1 Internal Medicine, Nova Southeastern University Dr. Kiran C. Patel College of Osteopathic Medicine, Clearwater, USA; 2 Internal Medicine, Nova Southeastern University Dr. Kiran C. Patel College of Osteopathic Medicine, Davie, USA; 3 Internal Medicine, Pioneer Medical Group, AdventHealth Ocala, Ocala, USA

**Keywords:** transesophageal echocardiogram, chronic pain management, disseminated bacteremia, acute pain management, methicillin-resistant staphylococcus aureus bacteremia

## Abstract

Methicillin-resistant *Staphylococcus aureus* (MRSA) is a bacteria that is present in both hospital and community settings. It commonly spreads through direct contact but may also spread through droplets. Our body’s innate and adaptive immunity is typically enough to protect against MRSA entering our body. MRSA has an increased ability to enter and spread throughout the body with the use of infected objects such as needles or even small breaks in the skin. When this spread occurs hematogenously, it is known as MRSA bacteremia. When a patient presents with MRSA bacteremia, it is a critical time-sensitive task to locate the source of infection as continual exposure to MRSA in the bloodstream can prolong infection and may ultimately be fatal. The interesting obstacle in our patient presenting with MRSA bacteremia was the inability to locate a source of infection, a pivotal component of medical management. After extensive testing and detailed history taking, there was no possible external source of infection, making source control a difficult task. Another unique component of this case report included the course of pain management and adjustments made to tailor pain control to this patient with a history of chronic opioid abuse.

## Introduction

Methicillin-resistant *Staphylococcus aureus* (MRSA) is a gram-positive bacteria that is present in both community and hospital settings. Most commonly, MRSA causes skin infections. There is increased risk in areas of overcrowding, increased skin contact, or having shared devices such as needles in the setting of intravenous (IV) drug abuse or shared personal items with those infected with MRSA [1]. Common symptoms to look out for in community-acquired MRSA skin infections include a bump or area of the skin that may have had a previous abrasion or small opening, which then progresses to redness, warmth, and swelling. In healthcare settings, however, an MRSA infection is much more serious. It can commonly cause pneumonia, bacteremia, or cellulitis. 

The clinical approach to MRSA bacteremia requires a strong patient history and detailed physical exam, and a well-equipped infectious disease team with several diagnostic imaging modalities [2]. The mortality of MRSA bacteremia may be quite high, up to 20-40% according to some studies. This makes it all the more imperative to assess patients critically [3]. Clinical manifestations of MRSA bacteremia include infective endocarditis (IE), indwelling device or prosthetic joint infection, sepsis, or osteomyelitis. Other rare manifestations of bacteremia include meningitis and bacteriuria. The typical workup for MRSA bacteremia begins with inquiring about potential sources of bacteremia. This includes, but is not limited to, indwelling catheters, joint hardware, and cardiac pacemakers. Other essential components of workup include consulting infectious disease, serial blood cultures until clearance of infection, transthoracic echocardiography (TTE) for IE evaluation, and further imaging based on the patient’s presenting complaints. 

The most important component of medical management is identifying the infection source and engaging in vigorous source control. Source control may include incision and drainage of the infected source or possibly removing an infected indwelling device. Serial blood cultures and susceptibilities are valuable for identifying which antibiotic therapies are best for eradicating the infection. For MRSA, the empiric antibiotic regimen includes IV vancomycin, with a loading dose of 20-35 mg/kg and then 15-20 mg/kg every 8-12 hours daily. As an alternative, IV daptomycin 6-10 mg/kg once daily can be considered [4]. The duration of IV antibiotic treatment depends heavily on the patient's comorbidities and complications of the bacteremia itself. Complications in managing MRSA bacteremia arise when a source is difficult to locate, as was the case with our patient. A more in-depth approach was needed at this time, with the goal of avoiding prolonged bacteremia and its associated unpleasant outcomes [5]. 

## Case presentation

We present a case of a 40-year-old Caucasian male, who presented to the emergency department (ED) with a two-week history of right leg pain. The pain was described as extending from the proximal posterior right thigh down to the right knee, without extending past the knee. The pain was described as a constant shooting pain that was 8/10 at its worst. Aggravating factors included active flexion of the knee, extension of the knee, and extension of the hip while the only alleviating factor was bedrest. The patient was unable to find a position of rest, straighten out his leg, or bear any weight at all. Worsening symptoms prompted the patient to come to the ED for further evaluation. When interviewed, the review of systems was negative, and the patient denied any recent history of falls, injury, or any straining events to the right leg. The patient also denied any loss of bowel, bladder function, or urinary retention. The patient’s past medical history was significant for percutaneous nephrolithotomy 10 years prior, which was incomplete, resulting in repeat lithotripsy and chronic lower back pain and repeat ED visits for associated pain. The patient had been seeing pain management physicians for the past 10 years and is a chronic user of narcotics. Other significant findings in past medical history include prior hospitalization for MRSA bacteremia of unknown source one month prior, which was complicated by Red Man Syndrome secondary to vancomycin administration and hospitalization for bacterial pneumonia three months prior. Family history was significant for lymphoma of unknown specifics, systemic lupus erythematosus, Crohn’s disease, and several other unidentified autoimmune diseases. The patient’s social history is significant for chronic tobacco and narcotic use. The patient did have a genetic spectrum screen done due to the presence of familial autoimmune conditions, but this did not reveal any autoimmune conditions. The initial labs and vitals taken at the ED were all within the normal range. The physical exam revealed an alert and well-oriented gentleman, who appeared uncomfortable and in pain. The musculoskeletal exam revealed normal range of motion, with equal strengths bilaterally, strong symmetric flexion, strong extension of ankle, knee, and hip areas as well as tenderness to palpation in the lower back region. A neurological exam revealed no focal neurological deficits, with intact cranial nerves, CN2 through CN12. The assessment made at the time was right-sided sciatica due to the overall clinical picture, history of disc disease, and history of spinal stenosis. The patient was given 10 mg of dexamethasone IV, Fentanyl 100 micrograms, along with ketorolac 30 mg IV and was discharged. The patient was given plans to follow up with a primary provider in two to three days, along with a course of non-steroidal anti-inflammatory drugs (NSAIDs) and crutches to assist with mobility. 

The patient returned to the ED one day later with continued complaints of severe proximal posterior right thigh pain. This pain had become much more aggravated that day and prompted him to revisit the hospital. The patient again reported similar symptoms of discomfort but denied any numbness, tingling, or weakness of the right lower extremity. A general physical exam revealed distress secondary to pain. At this time, the assessment of lumbar radiculopathy was made. The patient was strongly suggested to undergo magnetic resonance imaging (MRI), which the patient refused due to personal scheduling conflicts. A plan was made for the patient to seek an outpatient MRI service the following day, and the patient was discharged with pain control medications. The patient returned to the ED the following day and reported he had not followed up on the outpatient MRI referral. He reported that he was having continued uncontrolled pain in his right leg, despite the pain medications he was discharged with. The review of systems revealed tingling of the right lower extremity as well as significant diaphoresis. The physical exam revealed the patient was in moderate distress secondary to pain. Findings of musculoskeletal and neurological exams were within normal limits similar to the previous exams done during the previous ED visits. The vitals obtained during this visit revealed persistent sinus tachycardia with the heart rate consistently at 120 beats per minute. The patient’s vitals at this time included a respiratory rate of 18 breaths per minute, a temperature of 98.5 F, and blood pressure of 141/90 mm Hg. Initial labs were significant for an elevated white blood cell count of 15.8, lactic acid was within normal limits at 0.6 mmol/L, and his hemoglobin level returned at 11.9 g/dL, showing he was slightly anemic. At that time, the decision was made to admit the patient to the hospital for further observation, pain management, MRI test, and a neurosurgery evaluation for uncontrolled right leg pain. Once admitted, an infectious disease physician was consulted due to the patient’s recent history of hospitalization due to MRSA bacteremia. An MRI was ordered, blood cultures and white blood cell counts were being followed and IV daptomycin was started, at a dose of six mg per kilogram every 24 hours because of the patient’s leukocytosis and clinical presentation. Other interventions made at that time included the continuation of home medications for pain control, IV fluids as well as other relevant specialty consults. Neurosurgery was able to obtain older MRI results of the patient from two months prior. This imaging demonstrated mild degenerative changes with small disc protrusion on the lower left side at L1-L2 as well as a small disc protrusion on the left at L5-S1. There were no findings of discitis or osteomyelitis throughout the thoracic spine. A nuclear medicine white blood cell whole body scan with Ceretec also revealed no areas with abnormal radiotracer accumulation. 

**Figure 1:**
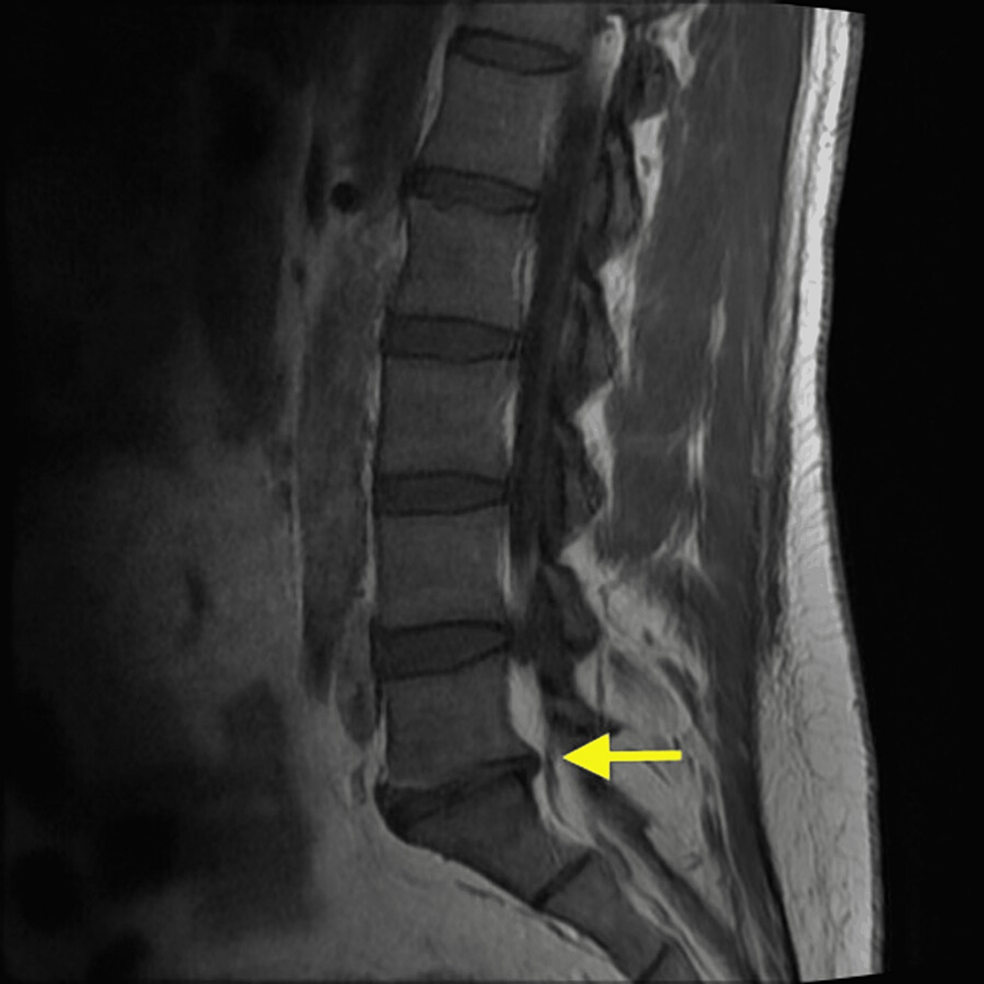
T1-weighted MRI lumbar spine without contrast demonstrated L5-S1 disc space narrowing with posterior disc bulge.

The neurosurgery team did not find any pathology of the lumbar spine that would warrant long-term opiate medication. Also, they did not find any evidence of radiculopathy. The MRI was unable to be completed given the patient’s pain and inability to lay flat for the procedure. The very limited study acquired sagittal T2-weighted images and concluded unlikely discitis and osteomyelitis, with L5-S1 disc space narrowing, posterior disc bulge, and mild canal narrowing (Figure 1).

**Figure 2:**
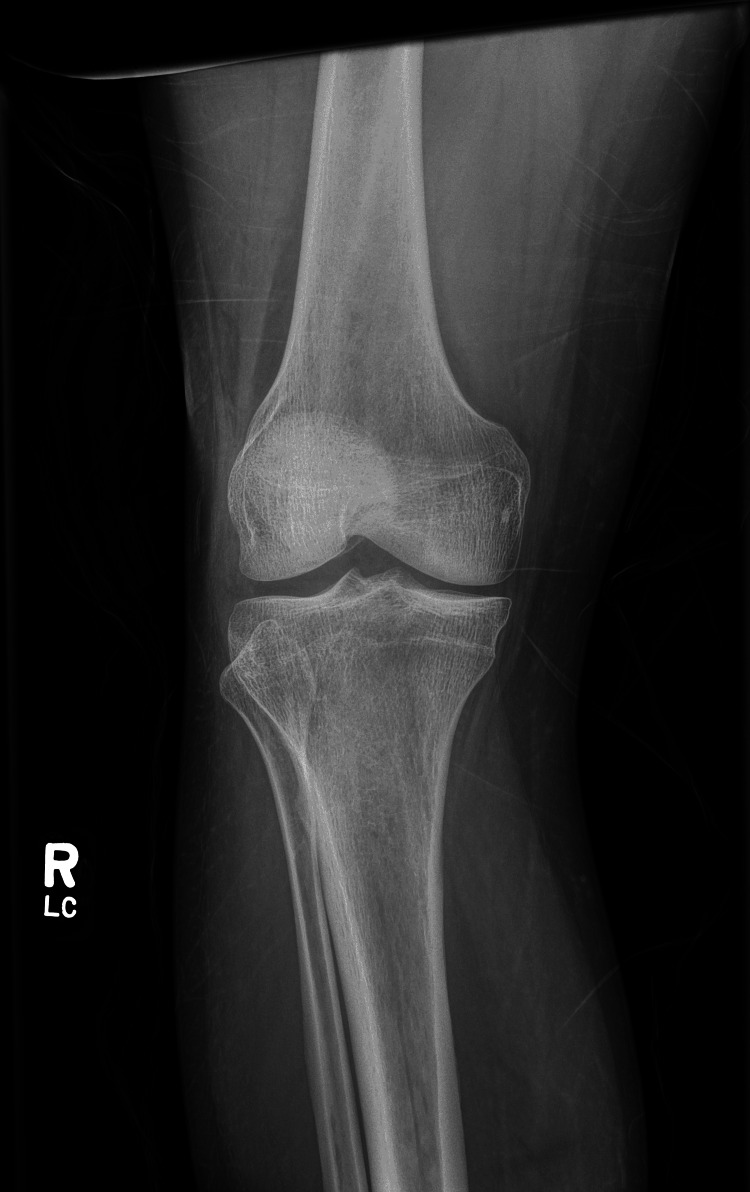
Anteroposterior (AP) x-ray of the right knee did not reveal any fractures or dislocations.

A lower extremity venous duplex ultrasound was negative for deep venous thrombosis. X-ray of the right knee showed some medial compartment joint space narrowing, but did not reveal any fractures or dislocations (Figure 2). Findings from a computed tomography angiography (CTA) of the chest included interval development of bilateral lung opacities with residual fine nodular opacity of the right upper lobe as well as splenomegaly. TTE showed an ejection fraction of 55-60%, with tricuspid valve and pulmonary valves not visualized well. Initial blood cultures came back positive for gram-positive cocci in clusters. This was later confirmed as *Staphylococcus aureus* positive for penicillin-binding protein (PBP2a) suggestive of MRSA. Further evaluation for right leg pain was continued with an orthopedic surgery specialist, who concluded there was no primary injury to the right knee or any signs of infection, and ruled out septic arthritis. A cardiology specialist was consulted for evaluation of possible IE, for which a transesophageal echocardiogram (TEE) was ordered. The results were negative for vegetation. 

**Figure 3:**
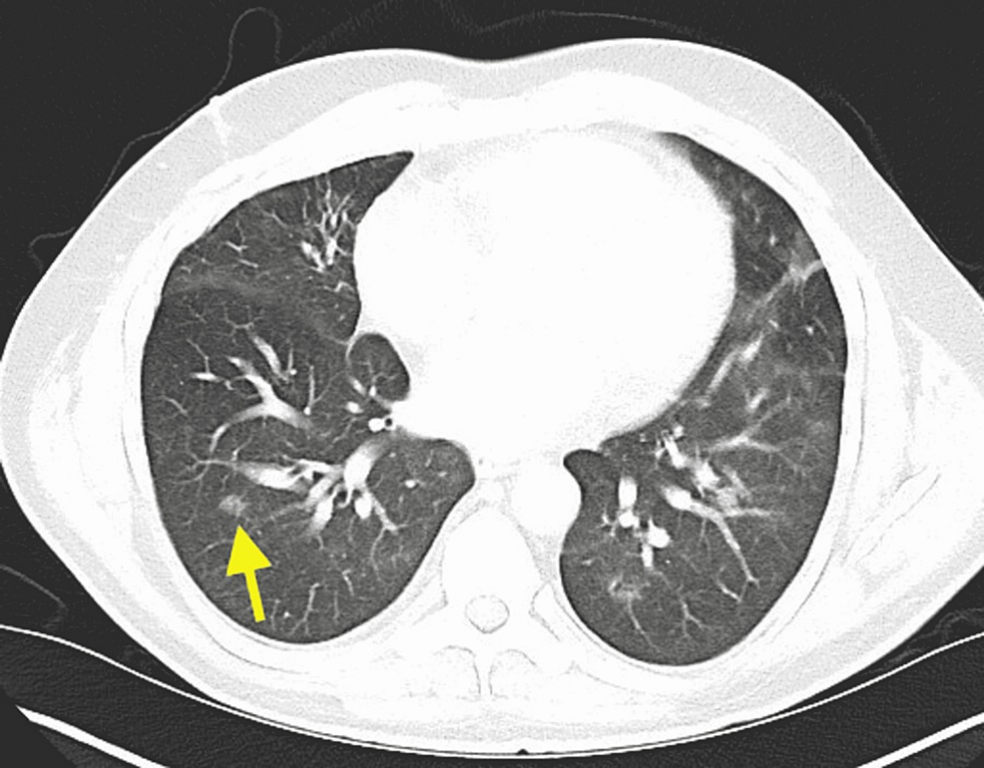
CT chest with contrast demonstrated nodular densities of the right lower lobe possibly representing septic emboli.

**Figure 4:**
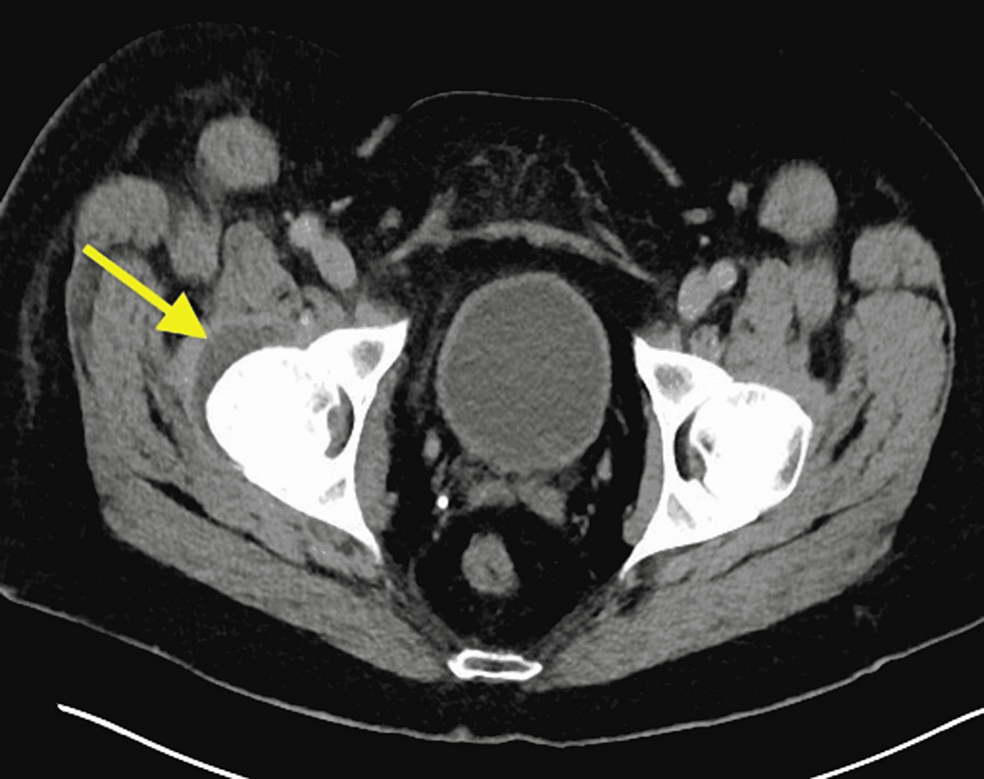
CT abdomen and pelvis with contrast demonstrated persistent right hip effusion.

On day six of admission, the patient had a fever of 104.6℉, and IV gentamicin 80 mg, every eight hours, was added to the existing antibiotic regimen of daptomycin for improved synergy. Blood cultures were still positive for MRSA, and susceptibility testing showed sensitivity to both vancomycin and gentamycin. At this point of the hospital course, there was still no identifiable primary source of MRSA. Because of this, a gallium scan and computed tomography (CT) scan was ordered to possibly identify a clear source of right hip pain. However, the patient continued to refuse the MRI due to his inability to lay flat for the test and stated he would only be able to do so under sedation. The providers then explained to the patient that such a procedure would not be feasible given the hospital’s protocol, which prohibited performing an MRI under sedation. The patient would require transfer to a tertiary care facility if he wished to have an MRI under sedation performed. CT of the chest with contrast revealed nodular densities of the right lower lobe possibly representing septic emboli in the setting of this patient’s bacteremia (Figure 3). CT of the abdomen and pelvis revealed fat stranding in the right lower extremity and persistent right joint effusion of the hip without any bony destruction (Figure 4). There was also reactive right pelvic adenopathy found on the scan. 

The Infectious Disease consultant’s differential diagnosis still included MRSA tricuspid valve endocarditis with septic emboli to the lungs and right hip. However, this was low on the list of possible diagnoses since the TEE was negative, and TEE for the diagnosis of valvular vegetations in endocarditis has a specificity of 91% and a sensitivity of 87% [6]. The team also suspected possible breakage of vegetation off the tricuspid valve prior to the TEE, which would explain the negative results. The plan at that time was to continue IV antibiotics and follow closely. By day 10 of admission, the patient reported improved hip mobility, decreased fevers, and was able to stand. Given the circumstances of several negative tests, the orthopedic surgery specialist recommended joint aspiration of the right hip to assess for cell count, culture, sensitivity, gram stainings, and crystals via an interventional radiology procedure. The culture of the right hip aspirate was also negative, but these findings may be due to prior antibiotic use for 10 days prior to the procedure and not being able to give the patient a significant antibiotic holiday. The results from the blood culture done on day 11 of admission came back negative, just like all of the previous cultures starting on day eight of admission, and the patient was prepared for discharge. The patient was discharged home with his IV port still in place, IV daptomycin, 650 milligrams every 24 hours for a total of six weeks, encouraged to ambulate, and was given precautions of when to return to the hospital for possible worsening symptoms or pain in the future. The patient was educated on the timeline for when to return for the removal of his IV port as well.

The tests that were performed during this patient’s hospital stay included a negative nuclear medicine white blood cell whole body scan with Technetium Tc 99m radiotracer, a negative lower extremity venous duplex ultrasound, a negative TTE study, a negative TEE, an x-ray of the right knee showing some medial compartment joint space narrowing, but no fractures or dislocations, a positive chest CTA that revealed interval development of bilateral lung opacities with residual fine nodular opacity of the right upper lobe as well as splenomegaly and a CT chest revealed nodular densities of the right lower lobe possibly representing septic emboli in the setting of bacteremia.

**Figure 5:**
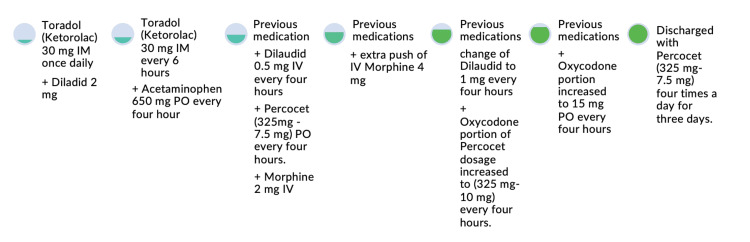
Summary of patient’s pain management course. PO: *per os* (by mouth)

With this report and patient presentation, we thought it pertinent to include the pain management course and changes that were made to the medication plan, especially considering the severe pain our patient was in (Figure 5). The patient’s home medications included duloxetine 30 mg twice daily, Percocet (acetaminophen-oxycodone) 325-750mg every four hours, indomethacin 25 mg three times daily, Robaxin (methocarbamol) 1500 mg three times a day, and Medrol Dosepak (methylprednisolone) 4 mg daily, but not all of these medications were part of his pain management plan during the hospital stay. The several changes made during the hospital stay were due to the patient’s continued pain despite the regimen that was placed. 

## Discussion

**Figure 6:**
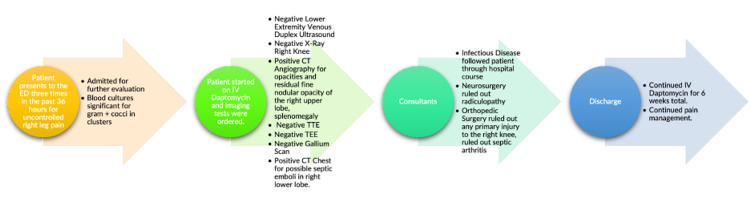
Summary of patient’s hospital course TTE: transthoracic echocardiogram; TEE: transesophageal echocardiogram

With the rise of MRSA infections in recent decades, it has become imperative to inform healthcare providers about both the common and rare presentations of this condition. In the case of our patient, where the source of infection was difficult to pinpoint, it led to frustration regarding decisions in management. The physicians followed an appropriate step-wise approach to treating this patient, with increased clinical suspicion given the patient’s recent hospitalization for MRSA and bacterial pneumonia (Figure 6). Repeat infection of *Staphylococcus aureus* within 30 days of the previous hospitalization is associated with increased mortality and longer hospital stays. Recurrence occurred in 22% of patients that survived a previous hospitalization for *Staphylococcus aureus *bacteremia and 17% more frequently in patients with MRSA bacteremia [7]. The patient presentation was unique in that there weren’t any major risk factors that are typically associated with MRSA bacteremia such as an indwelling prosthetic device like a vascular catheter or orthopedic prostheses. In addition, there was not any reported previous or current history of IV drug use. Other commonly associated risk factors of MRSA bacteremia like diabetes mellitus with poor glycemic control, chronic nasal colonization of *Staphylococcus aureus*, HIV infection, cellulitis, ulcers, or wounds commonly occurring in residents of long-term care facilities were also not present in our patient [8]. The only major risk factor that was present in the patient was the previous hospitalization for MRSA bacteremia of unknown source, which also had no major preceding risk factors. The positive blood cultures for *Staphylococcus aureus* with penicillin-binding protein (PBP2a) and previous knowledge that vancomycin caused Red Man Syndrome in the patient were the crucial guiding factors in taking care of this patient and deciding which medications would be appropriate. The likelihood of this MRSA bacteremia case being sourced from IV drug use was nullified with the history that was obtained. The rationale behind the several negative tests may have been due to the possible breakage of vegetation off the tricuspid valve prior to the TEE, resulting in a negative TEE. Another limitation is that the patient was not able to get an MRI study done, which could have potentially identified a source for his MRSA bacteremia not seen on CT scans.

Pain management is a key part of a physician’s duties. We are tasked with not only treating the disease process that is afflicting our patients but also preventing any undue suffering throughout this process. Here arises the dilemma, either being liberal with pain medications and eradicating all pain more definitively, or being more conservative and possibly only partially extinguishing the patient’s pain along with avoiding the long list of possible side effects associated with pain medications. There are many guidelines for appropriate pain management to help guide physicians in impartially choosing a path forward for their patients who present with drastic acute pain. These guidelines are evidence-based, and during the current devastating national opioid crisis in the United States, we so desperately need more physicians to educate themselves on appropriate pain management practices. Having one unified standard is a large part of the solution moving forward in this crisis. Physicians will not have to juggle with a guilty conscience about not prescribing certain medications when resorting to these objectives and evidence-based national guidelines for such practices.

One such example of a set of evidence-based guidelines for chronic pain management with opioid medications is the Centers for Disease Control and Prevention's Guideline for Prescribing Opioids for Chronic Pain, published in 2016 [9]. These guidelines detail the appropriate methodologies for assessing when it is appropriate to initiate opioid medications in patients with chronic pain, how to continually reassess these patients to make sure they still need these medications, and also how to assess for abuse of other substances or diversion in this patient population [9]. Patients with chronic pain are particularly vulnerable to opioid misuse, so this is a population that should be paid especially close attention to [10]. As far as opioid misuse is concerned, physicians may preemptively screen for these behaviors by checking Electronic-Florida Online Reporting Controlled Substance Evaluation (E-FORCSE) before writing any prescription for a controlled substance. The American Academy of Family Physicians recommends that physicians use their state’s Prescription Drug Monitoring Program prior to prescribing controlled substances, particularly opioids, to hopefully catch opioid abuse early, thus, affording the clinician the opportunity for a meaningful intervention [10].

In patients receiving chronic treatment with heavy regimens of opioids, these medications may lose efficacy at the same doses that would alleviate pain in non-tolerant individuals [11]. Pain management in patients such as ours is better suited to personalized regimens according to their tolerance and changing pain levels, as was the situation during this case’s hospital stay. The first of these suggestions involve maintaining opioid dosing at the minimum necessary to achieve the targeted pain level, potentially lessening the adverse effects associated with this class of medications [12]. In addition, a physician may elect for a technique termed opioid rotation where different opioids are prescribed in an alternating manner to decrease the chance of becoming too tolerant of one medication or non-responsive to another medication [13]. Adherence to these basic protocols will hopefully prevent patients from becoming reliant on opioids, avoiding long-term use of said opioids, and then avoiding illicit drug use. Research has shown a tendency for a certain proportion of people who are prescribed opioids to eventually progress to using heroin illicitly [14]. There is a significant association that exists between those who inject illicit drugs and the risk for invasive MRSA infections. IV drug users are greater than 16 times more likely to develop invasive MRSA infections [15]. 

## Conclusions

In conclusion, MRSA bacteremia should be extensively worked up in order to find a source of infection. In a patient with an indeterminate source, the patient should still be treated with broad spectrum antibiotics. It is important to utilize best-practice and evidence-based pain management strategies in order to reduce the likelihood of patient dependence. Further investigation could have been conducted for this patient. Although this patient refused an MRI of his hip due to his inability to lie down, accommodations with a tertiary care center would have allowed this patient to undergo sedation with close observation for the duration of imaging. An MRI would have given us a better clinical picture regarding which infectious process was taking place and given us insight to possible soft tissue pathologies. The use of facial CT may have eliminated possible periodontal abscess as a source of bacteremia. All things considered, the patient recovered lower extremity strength with the help of physical therapists and was discharged with pain medications and detailed instructions regarding his antibiotic course.
